# Bisphenols in Aquatic Products from South China: Implications for Human Exposure

**DOI:** 10.3390/toxics12020154

**Published:** 2024-02-16

**Authors:** Yinhai Chen, Xiurong Chen, Wenchi Lin, Jinghong Chen, Yuejun Zhu, Zhanghong Guo

**Affiliations:** Center for Disease Control and Prevention of Shantou, Shantou 515041, China; 13829450098@163.com (X.C.); zhguo2889@163.com (Z.G.)

**Keywords:** bisphenols, aquatic products, levels, risk assessment

## Abstract

In this study, 245 representative samples of aquatic products were selected from local markets in Shenzhen by stochastic sampling. The samples comprised eight species and fell into three aquatic product categories: fish, crustaceans, and bivalves. A total of eight BPs were determined by liquid chromatography coupled with mass spectrometry, namely, bisphenol A (BPA), bisphenol AF (BPAF), bisphenol AP (BPAP), bisphenol B (BPB), bisphenol S (BPS), bisphenol P (BPP), bisphenol Z (BPZ), and bisphenol F (BPF). All BPs were detected in aquatic products, except for BPAF, indicating pervasive contamination by BPs in aquatic products. BPS demonstrated the highest detection rate both before and after enzymatic hydrolysis, whereas BPAP exhibited the lowest detection rate before enzymatic hydrolysis and BPB displayed the lowest detection rate after enzymatic hydrolysis. The concentration difference before and after enzymatic hydrolysis proved to be statistically significant. Moreover, 49–96% of BPs in aquatic products were found in the combined state, underscoring the essentiality of conducting detections on aquatic product samples following enzymatic hydrolysis. While the health risks associated with ingesting BPs residues through aquatic product consumption were found to be minimal for residents at risk of exposure, the results suggest the necessity for more stringent regulations governing the consumption of aquatic products.

## 1. Introduction

Bisphenols (BPs) are recognized as an important class of synthetic chemicals that are widely used as raw materials to make epoxy resins and polycarbonate plastics. These materials are used in the manufacturing of a number of products, such as food and drink containers, thermal paper receipts, and baby products [1,2,3]. Polycarbonate plastic production involves synthesizing bisphenol A (BPA). Polycarbonate plastics are popular among manufacturers worldwide due to their economic viability, color adaptability, thermal stability, and corrosion resistance. This popularity is reflected in the annual production of approximately 5 million tons of BPA [4]. It has been reported that BPA has been found in a wide range of environmental components, including surface water, drinking water, soil, and sediment [5,6,7,8]. Humans are exposed to BPA via ingestion, inhalation, and dermal absorption through different sources. BPA is classified as an endocrine-disrupting chemical (EDC) due to its estrogenic and antiandrogenic properties, raising concerns about its potential impact on human health. Moreover, BPA is linked to various adverse health effects, including obesity, cardiovascular disease, cognitive decline, diabetes, and reproductive defects [9,10,11,12,13].

Due to restrictions on the use of BPA in various countries, alternative compounds called BPA analogs have been developed as substitutes. Examples of these analogs include Bisphenol AF (BPAF), bisphenol AP (BPAP), bisphenol B (BPB), bisphenol S (BPS), bisphenol P (BPP), bisphenol Z (BPZ), and bisphenol F (BPF). However, studies on the toxicity of these analogs have shown that they have antiandrogenic and estrogenic properties that are comparable to, if not more potent than, those of BPA [14,15]. As an illustration, a study conducted on young female rats showed that BPS could affect genes linked to the dopamine and serotonin systems, as well as 5α-reductase in the prefrontal cortex [16]. According to Ullah et al., adult rats exposed to BPB experience oxidative stress in the testes, which leads to a decrease in daily sperm viability and production [17]. Additionally, maternal exposure to BPF has been found to increase anxiety and depressive behaviors in the offspring of mice [18]. Moreover, BPAF induces inhibition of testosterone production by altering genes and proteins in the testosterone biosynthesis pathway in adult male rats [19]. As a result, the extensive usage and environmental persistence of these compounds pose a significant concern for public health due to their potential impact on biological systems.

Aquatic products, including fish, shrimp, shellfish, etc., are distinguished by their delightful taste and substantial nutritional value. Given these attributes, they have evolved into essential sources of crucial nutrients such as vitamins, protein, and trace elements [20]. Aquatic products are a major source of animal protein for residents living in coastal areas. China’s demand for aquatic product consumption has been rising recently [21]. Between 2008 and 2019, China’s annual consumption of aquatic products averaged 53 million tons, as reported. Nevertheless, these products may harbor heavy metals or other emerging organic pollutants (EOPs) like BPs, parabens, and polychlorinated biphenyls (PCBs). The entry of these pollutants into aquatic products can occur through diverse pathways, encompassing water, sediment, aquaculture, transportation, processing, and storage [21,22]. These contaminants, which are highly concentrated in aquatic products, progressively build up through the food chain and food web and have a negative impact on human health. Numerous countries, including China, have established stringent limit standards for substances universally recognized as harmful to human health. However, no country has instituted definitive guidelines for certain emerging pollutants, like BPs. Consequently, accurately measuring the content of BPs in aquatic products becomes imperative for relevant regulatory authorities to establish safety dosage limits for associated food items.

Situated as a coastal city in South China, Shenzhen hosts several aquaculture bases. The economic development of Shenzhen has inevitably led to environmental pollution, which is exacerbated by human activities that may contribute to contamination. This contamination poses potential health impacts for residents who consume aquatic products in the region. To fill the current data gap, 245 representative aquatic product samples in Shenzhen were collected stochastically from local markets. The samples included eight species and three aquatic product categories: fish, crustaceans, and bivalves. High-performance liquid chromatography–tandem mass spectrometry (HPLC-MS/MS) was used to detect eight BPs (BPA, BPAF, BPAP, BPB, BPS, BPP, BPZ, and BPF) from aquatic products. In this paper, we aim to achieve the following objectives: (1) quantify BP levels in aquatic products; (2) investigate the factors affecting BP levels; and (3) estimate the human health risks associated with aquatic product consumption.

## 2. Materials and Methods

### 2.1. Reagents and Materials

BPA, BPAF, BPAP, BPB, BPS, BPP, BPF, BPZ, ^13^C_12_-BPA, ^13^C_12_-BPS, ^13^C_12_-BPAF, ^13^C_12_-BPB, and BPF-d_10_ were purchased from Toronto Research Chemicals (Toronto, ON, Canada). Methanol, acetonitrile, n-hexane, and β-glucuronidase (aqueous solution > 100,000 units/mL, sulfatase activity < 20,000 units/mL) were purchased from Shanghai Anpu Experimental Technology (Shanghai, China). Acetic acid and ammonium acetate were purchased from Shanghai Jingchun Biochemical Technology (Shanghai, China). Solid-phase extraction (SPE) cartridges (Oasis PRiME HLB, 200 mg/6 mL) were purchased from Waters (Framingham, MA, USA). Ultra-pure water was provided by the Millipore water purification system (Billerica, MA, USA).

### 2.2. Sampling and Sample Preparation

To mitigate any potential sampling bias, a total of 245 representative aquatic product samples were stochastically collected from local markets in Shenzhen. The samples represented three aquatic product categories, namely, fish (including six species—*Oreochromis mossambicus*, *Lateolabrax maculatus*, *Carassius auratus*, *Ctenopharyngodon idella*, *Aristichys nobilis* and *Trachinotus ovatus*, *n* = 171), crustaceans (one species of *Metapenaeus ensis*, *n* = 56), and bivalves (one species of *Paphia undulata*, *n* = 18). After the edible parts were homogenized, the samples were stored in airtight bags at −20 °C until further analysis.

For the enzymatic group samples, 2.0 g samples were weighed, then 50 μL mixed internal standard was added. The pH was adjusted with 3 mL 0.1 mol/L ammonium acetate buffer and an additional 20 μL β-glucuronidase. Samples were shaken at 37 °C for 12–16 h, avoiding light. Then, 9 mL acetonitrile was added for extraction, followed by shaking at 2500 rpm (the relative centrifugal force, RCF: 560× *g*) for 5 min, and 10 min ultrasonication. The samples were centrifuged at 9000 rpm (RCF: 7258× *g*) for 5 min, and the process was repeated twice. After supernatant removal and addition of 2 mL n-hexane, the samples were shaken for 45 s and centrifuged at 9000 rpm (RCF: 7258× *g*) for 5 min, then the lower phase was obtained and passed through the solid-phase extraction (SPE) column. Next, 2.5 mL of the extract was blown nearly dry with nitrogen and redissolved in 60% methanol solution. Finally, it was filtered through a 0.22 μm membrane for instrumental analysis.

The procedure for samples without enzymatic digestion was similar to those with enzymatic digestion. A volume of 50 μL mixed internal standard was added to a 50 mL pointed bottom centrifuge tube containing 2.0 g of aquatic product samples, and then 10 mL acetonitrile was added for extraction. The following steps were the same as those described for the enzymatic group samples.

### 2.3. Instrumental Analysis

Target chemicals were analyzed using a 20A HPLC system (Shimadzu, Tokyo, Japan) coupled with a Q-Trap 5500 tandem mass spectrometer (MS/MS; Applied Biosystems, Foster City, CA, USA). Chromatographic separation of the analytes was performed on an Atlantis C18 column (1.7 μm, 3.0 × 100 mm, Waters, Dublin, Ireland), accompanied by mobile phases of water and methanol. The gradient elution conditions and the mass spectrometric information of the target chemicals are shown in Tables S1 and S2, respectively. The injection volume was 5 µL and the column temperature was set at 40 °C. The flow rate was set at 0.3 mL/min. The mass spectrometer was performed in negative electron spray ionization (ESI) mode. The source temperature was set as 550 °C and the ionization voltage was −4500 V.

### 2.4. Quality Assurance and Quality Control (QA/QC)

To show potential contamination during the experiment, a blank sample was analyzed every 25 samples. No target compounds were detected in the blanks. The linear range of all target chemicals was 0.1–50 μg/L with the correlation coefficient (*R*^2^) being above 0.99. The limits of detection (LOD), defined as the signal-to-noise (S/N) ratio = 3, ranged from 0.002 to 0.09 ng/g and the limits of quantification (LOQ), defined as the S/N ratio = 10, ranged from 0.007 to 0.03 ng/g. The recoveries were between 84.8% and 111%. The reaction stability of the instrument was confirmed by 100 μg/L mixed internal standard solution. The LODs, LOQs, and recoveries of the applied analytical methods are described in detail in Table S3.

### 2.5. Health Risk Assessments

The estimated daily intake (EDI) of BPs due to consumption of aquatic products was calculated according to the following Equation (1):

(1) $$EDI=\frac{C_{a}\times CR}{BW}$$

where *C_a_* (ng/g ww) is the concentration of various BPs in aquatic products. *CR* (g/day) is the daily consumption of aquatic products, and according to the China Fishery Statistical Yearbook 2020, a typical Chinese resident consumed aquatic products at a rate of 76.16 g/day in 2019. *BW* (kg) is the average body weight of the population, which is on average 63.9 kg for males and 54.0 kg for females according to the *Report on the Nutrition and Chronic Disease Status of Chinese Residents 2020*.

The hazard quotient (HQ) was used to preliminarily assess the health risk of exposure to BPs through a single route. The value can be obtained through the following Equation (2):

(2) $$HQ=\frac{EDI}{ADI}$$

where EDI (ng/kg bw/day) is the estimated daily intake of each BP calculated previously; ADI (ng/kg bw/day) is the allowable daily intake; and 50 μg/kg bw/day was assigned according to European Food Safety Authority (EFSA) and U.S. Environmental Protection Agency (EPA).

### 2.6. Data Analysis

Statistical analysis was performed using Excel 2016, IBM SPSS 26.0, and Python 3.7. Descriptive statistics, including mean, median, maximum, and minimum values, were employed to characterize the concentration of BPs in aquatic products. Pearson’s correlation coefficient (bilateral) test or Spearman’s rank correlation test were used to analyze the correlation between various substances. Inter-group differences between enzymatic and non-enzymatic hydrolysis were examined using the Mann–Whitney U test. Statistical significance was established at *p* < 0.05.

## 3. Results and Discussion

### 3.1. Levels of Bisphenols in Aquatic Products

Table 1 displays the detection rates and concentration levels of BPs in aquatic products sold in Shenzhen. The concentration and detection rates of BPs before enzymatic hydrolysis were 0.08–94.1 ng/g ww and 6.12–78.8%, respectively. Following enzymatic digestion, the samples showed different detection rates of BPs, from 33.9% to 97.1%, and the concentrations of BPs, from 0.01 ng/g ww to 162 ng/g ww. These results indicated that the aquatic products were found to be generally contaminated with BPs, and BPS had the highest detection rate both before and after enzymatic digestion. The highest detection rate for BPS (50.8%) was also observed in the muscle tissue of fish from distinct aquatic environments [23]. BPAF was essentially undetectable, regardless of enzymatic digestion. The BPA and BPF concentrations after enzymatic digestion in this experiment ranged from 0.01 to 19.9 ng/g ww and from 0.20 to 138 ng/g ww, respectively. The levels of BPA in marine and freshwater fish from Hong Kong (0.31–19.25 ng/g ww) were comparable to the results after enzymatic digestion in this study. However, the BPF levels (2.30–26.37 ng/g ww) were considerably lower compared to those observed post enzymatic digestion [24]. The mean concentration of BPA in mollusks from the Bohai Sea recorded at 1.03 ng/g ww, and the mean concentration of BPF at 0.747 ng/g ww, were both lower than the outcomes of the current study [25]. In contrast, the BPA levels found in adult shad from the Ganges River, India, ranging between 172 and 288 ng/g ww, significantly exceeded the results of the present study [26].

Table S4 presents the outcomes of the comparative analysis of enzymatically and non-enzymatically dissolved BPs. Except for BPB (*p* > 0.05), the concentrations of all BPs in enzymatically and non-enzymatically dissolved states exhibited statistically significant differences (*p* < 0.05). The mean concentrations of BPs increased by a factor of 1.97 to 26.25 from pre-treatment levels. This observation is likely attributed to the action of β-glucuronidase/sulfate lyase [27], which hydrolyzes bound BPs in the organism, thereby transitioning it to the free state, resulting in elevated concentrations as observed in the results. The bound state content of BPs in aquatic products ranged from 49.0% to 96.0%, as depicted in Figure S1. Elevated concentrations and detection rates after enzymatic digestion indicated a higher proportion of bound BPs in aquatic products. Various studies have utilized comparable hydrolysis techniques to explore the BPs content in aquatic products. Wong et al. (2017) identified BPA concentrations in the free state ranging from 0.83 to 19.25 ng/g across 20 fish species, a range consistent with the results of the current study [24]. In a study by Wang et al. (2020), the investigation into eight distinct BP types in carp revealed proportions in the free state ranging from 21% (BPS) to 48% (BPAP) [28]. Conversely, the present study indicated that the proportions of BPA, BPS, and BPB in the fish were higher compared to their investigation. The metabolic pathways of BPs in organisms exhibit considerable variation across different sources of exposure [29]. Furthermore, the specific type of aquatic product and variations in conditions such as temperature, as well as distinctions between marine and freshwater environments, may further influence the metabolism of these substances in organisms.

### 3.2. Compositional Profiles and Potential Sources

**Figure 1:**
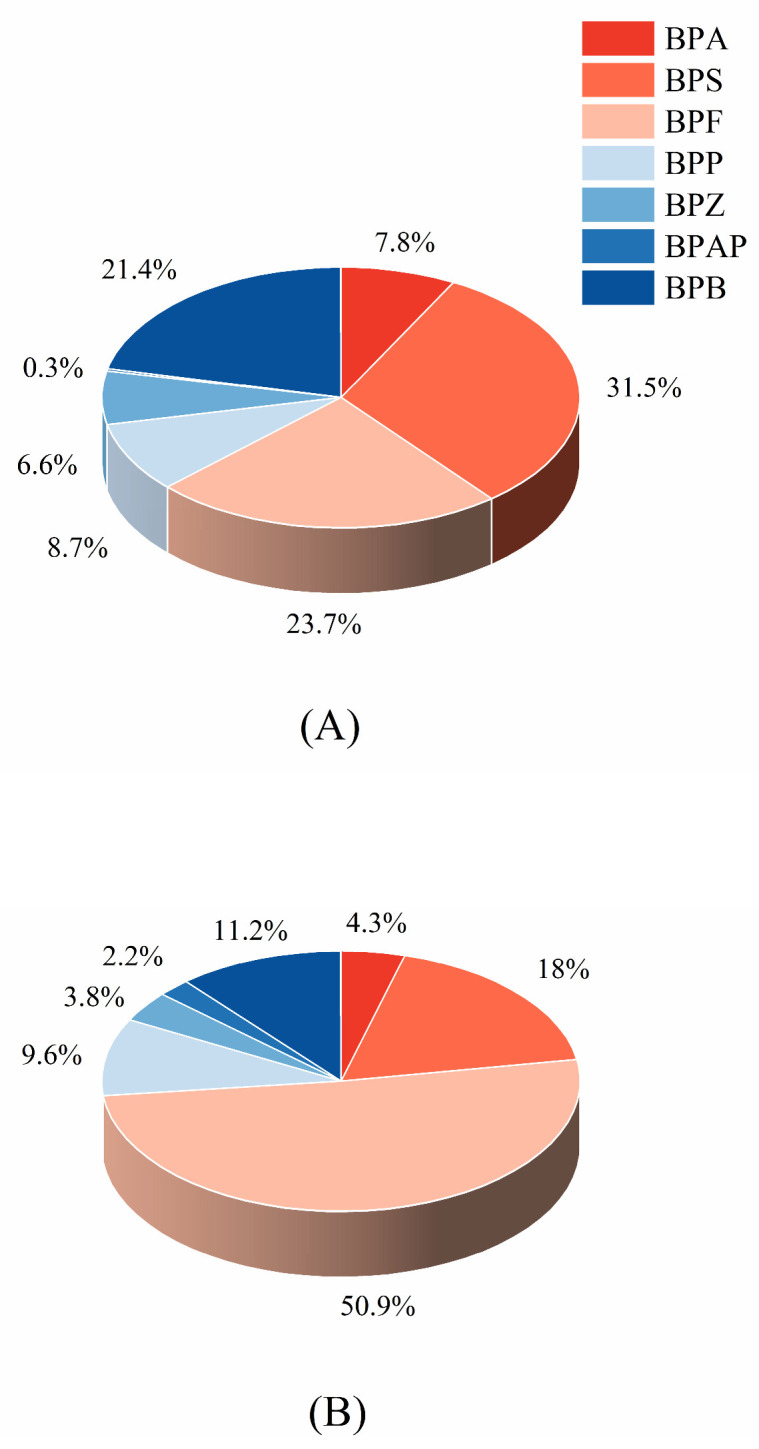
Compositional profiles of BPs in non-enzymatic samples (**A**) and enzymatic (**B**) aquatic products.

Figure 1 illustrates the composition profiles of detected BPs in aquatic products both without and with enzymatic digestion. In the non-enzymatic samples, the highest concentration was attributed to BPS, constituting 31.4% of the total free-form BPs, followed by BPF (23.7%), BPB (21.4%), BPP (8.7%), BPA (7.8%), BPZ (6.6%), and BPAP (0.3%), respectively. As for the enzyme-treated aquatic samples, BPF emerged as the predominant contributor in both free and bound states, accounting for 50.9% of the total BPs, followed by BPS (18%), BPB (11.2%), BPP (9.6%), BPA (4.3%), BPZ (3.8%), and BPAP (2.2%), respectively. It is noteworthy to mention that a considerable proportion of the composition in both enzymatically and non-enzymatically digested samples is composed of the combination of BPF and BPS, as opposed to the conventional assumption that the highest levels are linked to BPA, which has been reported in the majority of studies [30]. Recent studies have revealed that BPA is gradually being replaced by BPS and BPF in various manufacturing processes, leading to their high prevalence in aquatic environments. This trend is reflected in the gradual decline of BPA concentration, with its analogs, particularly BPS and BPF, occupying an increasingly substantial portion of the aquatic environment [31]. Some studies even found that the concentration of BPS can be equal to that of BPA [32], and higher concentrations of BPF in surface waters may be anticipated in the near future [33]. The content of BPs in organisms is not only related to exposure but may also be affected by metabolism. In subsequent studies, more attention should be paid to the aquatic environment and organisms.

**Figure 2:**
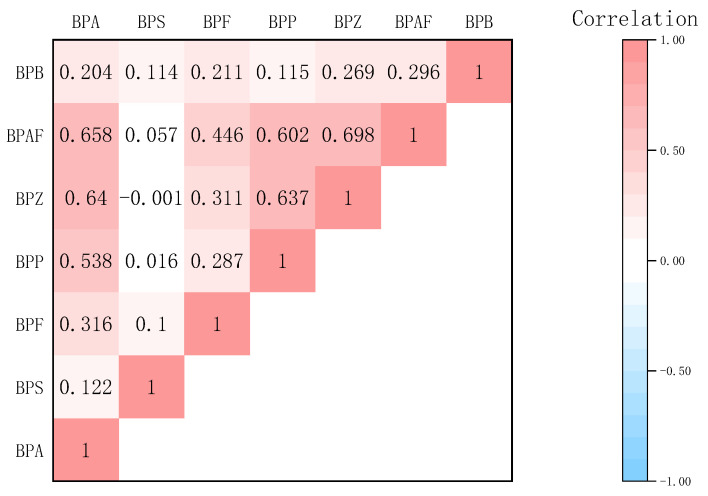
Spearman’s correlation between pairs of BPs in aquatic products.

For a more in-depth examination of the sources of BPs, pairs of the identified BPs in enzymatically digested samples were analyzed using Spearman’s correlation (*p* < 0.05), as illustrated in Figure 2, with the correlation coefficients ranging from 0.001 to 0.698. The results of the analysis reveal a statistically significant positive association between BPB and BPA, BPF, BPP, BPZ, and BPAP. However, no significant correlation was observed between BPS and other BPs, implying a divergence in their sources. Moreover, a strong positive correlation was identified between BPA and BPAP, BPZ, BPP, and BPF. Notably, the relatively high correlation coefficients for BPF-BPAP, BPP-BPZ, and BPP-BPAP suggest the possibility of common sources, such as sewage treatment plants, municipal solid waste, and landfill leachate [34]. The addition of different analogs to goods in place of BPA might have contributed to the aforementioned findings. Therefore, further research is needed to identify independent sources of BPA analogs.

### 3.3. Differences in Bisphenols Levels across Types of Aquatic Product

**Figure 3:**
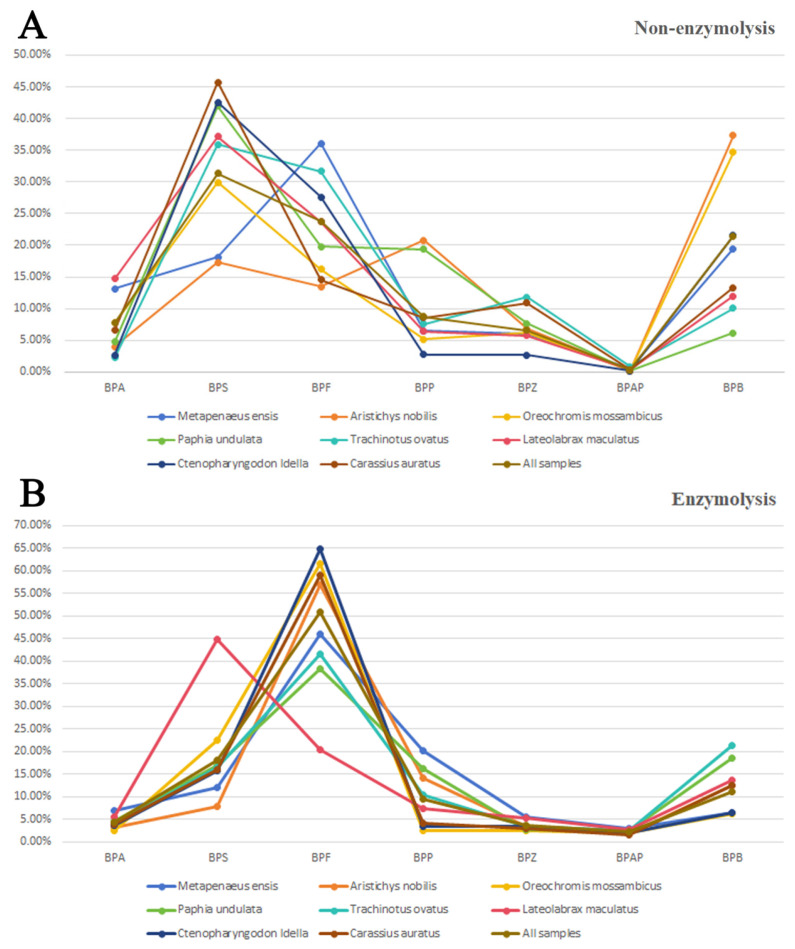
Distribution of BPs in different aquatic products. (**A**) Distribution of BPs in different aquatic products without enzymolysis; (**B**) distribution of BPs in different aquatic products with enzymolysis.

The distribution of BPs in different aquatic products is shown in Figure 3. In different aquatic products, the concentration ranking from high to low before enzymatic hydrolysis is as follows: *Aristichys nobilis*, *Oreochromis mossambicus*, *Ctenopharyngodon idella*, *Lateolabrax maculatus*, *Paphia undulata*, *Carassius auratus*, *Metapenaeus ensis,* and *Trachinotus ovatus*. After enzymatic hydrolysis, the concentration ranking changes to *Carassius auratus*, *Aristichys nobilis*, *Ctenopharyngodon idella*, *Oreochromis mossambicus*, *Paphia undulata*, *Trachinotus ovatus*, *Metapenaeus ensis*, and *Lateolabrax maculatus*. The analysis indicates a consistently high concentration of BPs in samples of *Aristichys nobilis* and *Ctenopharyngodon idella*. Before enzymatic hydrolysis, BPS exhibits the highest concentration in samples except for *Oreochromis mossambicus*, *Aristichys nobilis*, and *Metapenaeus ensis*, where a high concentration of BPB or BPF is observed. After enzymatic hydrolysis, BPF emerges as the compound with the highest proportion of BPs in samples, except for *Lateolabrax maculatus*, which is still rich in BPS.

In contrast to our findings, previous studies have only detected BPA in fish muscles, with an average concentration of 16.2 ng/g. BPB was detectable in fish livers, with an average concentration of 7.3 ng/g. However, BPF was not detected in any of the samples [35]. Aligning more closely with our study, Rios-Fuster et al. observed that BPA exhibited the highest concentration in fish, followed by BPS and BPF [36]. The concentration of BPA in shrimps shows correspondence between our study (*Metapenaeus ensis*: 2.49 ng/g) and another study located in Pearl River Estuary (*Penaeuschin ensis*: 0.16–4.36 ng/g) [37]. The concentration of BPA (1.63 vs. 1.85 ng/g) and BPB (6.62 vs. 7.22 ng/g) in clams, as reported by Antía Lestido-Cardama, aligns closely with our study. However, the concentration of BPF (14.97 vs. 1.52 ng/g) in their study is approximately ten times lower than observed in our investigation [38]. In a separate study, it was noted that the average concentration of BPA in Rangia cuneata, a type of bivalve, was 174 ng/g, which is approximately 100 times higher than the concentration observed in *Paphia undulata* in the current study [39]. It is evident that the concentration and distribution of BPs in sea products exhibit significant variations across different areas.

### 3.4. Exposure Risk Assessment

The EDIs of ∑_7_BPs (the sum concentration of all the studied BPs) in different aquatic products are shown in Table 2. The 95% quantiles of EDI were in the range of 63.76–157.4 ng/kg bw/day in males and 75.45–186.3 ng/kg bw/day in females. In the consumption of identical types of aquatic products, except for *Trachinotus ovatus*, females exhibited greater EDI compared to males. The highest EDI, with a 95% quantile, is associated with the consumption of *Carassius auratus*, followed by *Oreochromis mossambicus*, *Aristichys nobilis*, *Ctenopharyngodon idella*, *Paphia undulata*, *Metapenaeus ensis*, *Lateolabrax maculatus,* and *Trachinotus ovatus*, which indicated that the intake of BPs varies across different aquatic products.

To further evaluate human exposure risks via aquatic product consumption, the HQ value was calculated using 95% quantile of the EDI value of aquatic products consumed by residents (Table 2). The HQ ranged from 0.0012 to 0.0031 for males and from 0.015 to 0.037 for females. Residents who consume *Carassius auratus* run a higher risk of BPs exposure compared to those who consume other aquatic products, as evidenced by the significantly higher HQ value of this species (*p* < 0.05). Females have a higher risk of BPs exposure than males, as indicated by the higher HQ value. The present study employed a relatively high EDI value and assumed a 100% absorption rate of BPs in the human body. It is noteworthy that the potential reduction in BPs content during food preparation was overlooked, leading to possible overestimation of human exposure to BPs. Despite these limitations, the calculated HQ values remained well below one, signifying that there is no significant health risk associated with the consumption of aquatic products by the residents.

## 4. Conclusions

In this study, a comprehensive understanding of the detrimental effects of BPs on organisms, as well as their direct or indirect impacts on humans, can be obtained by systematically measuring and analyzing their levels in aquatic products. The analysis of 245 aquatic products sold in Shenzhen revealed detectable levels of BPs, and the associated risks for residents by gender were assessed. All BPs were present in aquatic products, except for BPAF, indicating widespread contamination. BPS exhibited the highest detection rate, while BPAP had the lowest rate before enzymatic hydrolysis, and BPB had the lowest rate after enzymatic hydrolysis. The concentration difference before and after enzymatic hydrolysis was statistically significant. Notably, 49–96% of BPs in aquatic products existed in a combined state, underscoring the importance of enzymatic hydrolysis in sample detection. There was a significant correlation between different BPs, with the exception of BPS, suggesting possible shared sources. The risks associated with male and female residents’ consumption of aquatic products containing BPs residues were negligible. However, the results highlight the need for stricter regulations regarding the intake of aquatic products. To reduce the human health risks associated with BPs, several suggestions were proposed, such as a systematic investigation of BPs’ fate in environmental media, the formulation of discharge standards for BPs pollution in industrial wastewater, and implementation of BP remediation projects.

## Figures and Tables

**Table 1:** Detection rate and content level of BPs in aquatic products (ng/g ww).

Sample	Process	Parameters	BPA	BPS	BPF	BPP	BPZ	BPAP	BPB
*Metapenaeus ensis*	Non-enzymolysis	DR (%)	42.9	82.1	42.9	23.2	16.1	5.60	25.0
		Median	0.36	0.80	2.54	0.63	0.47	0.06	1.15
		Mean	1.29	1.78	3.54	0.64	0.60	0.05	1.91
		Minimum	0.01	0.02	1.05	0.04	0.04	0.01	0.09
		Maximum	9.84	12.7	15.8	1.73	1.73	0.07	11.9
	Enzymolysis	DR (%)	69.6	98.2	51.8	53.7	82.1	69.6	23.2
		Median	2.16	1.76	7.32	3.37	1.54	1.28	0.36
		Mean	2.49	4.34	16.7	7.30	2.04	1.11	2.34
		Minimum	0.31	0.03	0.26	0.01	0.01	0.01	0.19
		Maximum	8.25	26.5	93.1	37.5	8.79	3.12	18.2
*Aristichys nobilis*	Non-enzymolysis	DR (%)	33.3	94.4	27.8	27.8	11.1	5.56	22.2
		Median	0.24	2.39	2.20	0.68	1.24	0.04	6.48
		Mean	0.73	3.13	2.44	3.77	1.24	0.04	6.76
		Minimum	0.05	0.13	0.49	0.24	0.44	0.04	0.36
		Maximum	3.39	9.66	4.46	15.2	2.03	0.04	13.7
	Enzymolysis	DR (%)	83.3	100	38.9	50.0	66.7	72.2	38.9
		Median	1.33	4.36	42.5	5.81	2.16	1.35	10.2
		Mean	1.85	4.58	33.2	8.27	2.05	1.04	7.23
		Minimum	0.05	0.48	3.37	1.56	0.33	0.03	0.18
		Maximum	7.47	10.5	66.8	16.9	3.08	1.63	11.9
*Oreochromis mossambicus*	Non-enzymolysis	DR (%)	34.3	68.7	31.4	14.3	11.4	2.90	37.1
		Median	0.19	4.79	1.88	0.47	0.47	0.03	0.95
		Mean	1.24	4.80	2.60	0.83	0.99	0.03	5.56
		Minimum	0.02	0.72	0.25	0.18	0.03	0.03	0.00
		Maximum	8.23	9.35	7.24	2.29	3.01	0.03	33.7
	Enzymolysis	DR (%)	65.7	97.1	40.0	22.8	51.4	51.4	22.8
		Median	0.97	5.20	26.9	0.96	1.28	1.31	2.71
		Mean	1.19	11.0	30.2	1.29	1.22	1.00	3.08
		Minimum	0.04	1.38	0.25	0.56	0.08	0.01	0.47
		Maximum	2.99	161	74.3	2.91	2.62	1.43	7.80
*Paphia undulata*	Non-enzymolysis	DR (%)	44.4	72.2	33.3	27.8	16.7	5.56	33.3
		Median	0.44	4.42	2.43	0.86	0.60	0.03	0.42
		Mean	0.52	4.55	2.15	2.10	0.83	0.03	0.67
		Minimum	0.14	1.89	0.36	0.15	0.01	0.03	0.09
		Maximum	1.37	8.60	4.29	7.17	1.87	0.03	1.64
	Enzymolysis	DR (%)	88.9	94.4	50.0	72.2	72.2	72.2	27.8
		Median	1.70	4.30	8.45	6.98	1.31	1.38	8.67
		Mean	1.85	6.66	15.0	6.31	1.09	1.00	7.22
		Minimum	0.07	1.28	0.42	0.54	0.33	0.10	0.30
		Maximum	6.01	29.9	49.1	12.3	2.07	1.62	9.59
*Trachinotus ovatus*	Non-enzymolysis	DR (%)	18.7	93.7	68.7	43.7	18.7	6.25	31.2
		Median	0.17	3.09	3.38	0.41	0.43	0.08	0.99
		Mean	0.22	3.47	3.06	0.73	1.14	0.08	0.97
		Minimum	0.04	0.34	0.41	0.08	0.03	0.08	0.04
		Maximum	0.44	8.48	6.30	2.97	2.97	0.08	1.81
	Enzymolysis	DR (%)	75.0	100	43.7	62.5	68.7	62.5	31.2
		Median	1.06	4.41	4.49	1.20	1.34	1.25	3.39
		Mean	1.48	6.19	15.8	3.98	1.33	0.94	8.09
		Minimum	0.25	2.59	0.70	0.13	0.03	0.01	0.43
		Maximum	4.81	19.4	78.6	20.9	2.96	1.60	19.2
*Lateolabrax maculatus*	Non-enzymolysis	DR (%)	35.3	82.3	50.0	14.7	14.7	2.94	23.5
		Median	0.33	2.61	2.48	0.88	0.85	0.04	0.45
		Mean	1.73	4.35	2.78	0.75	0.67	0.04	1.40
		Minimum	0.02	0.01	0.06	0.38	0.27	0.04	0.10
		Maximum	9.30	25.0	8.45	0.99	0.96	0.04	5.55
	Enzymolysis	DR (%)	52.9	97.0	32.3	41.2	44.1	50.0	38.2
		Median	1.32	6.21	4.03	1.95	1.84	1.31	5.16
		Mean	1.85	15.1	6.89	2.47	1.77	0.93	4.59
		Minimum	0.00	0.63	0.71	0.35	0.11	0.01	0.14
		Maximum	5.19	122	30.7	6.45	3.42	1.85	12.4
*Ctenopharyngodon idella*	Non-enzymolysis	DR (%)	33.3	69.4	55.6	19.4	16.7	8.33	47.2
		Median	0.19	2.56	3.23	0.19	0.22	0.03	1.65
		Mean	0.40	6.30	4.09	0.41	0.40	0.03	3.19
		Minimum	0.02	0.06	0.40	0.01	0.01	0.02	0.02
		Maximum	2.27	94.1	21.0	1.32	1.32	0.03	16.8
	Enzymolysis	DR (%)	72.2	94.4	30.6	47.2	61.1	72.2	41.7
		Median	1.67	4.20	25.2	1.21	1.99	1.26	1.63
		Mean	1.99	8.48	34.7	1.84	1.86	1.07	3.52
		Minimum	0.11	0.36	1.44	0.04	0.51	0.01	0.18
		Maximum	7.44	74.5	138	5.39	3.52	2.09	15.3
*Carassius auratus*	Non-enzymolysis	DR (%)	50.0	78.1	43.7	31.2	28.1	12.5	50.0
		Median	0.20	2.68	0.91	0.46	0.44	0.04	1.09
		Mean	0.70	4.87	1.55	0.91	1.16	0.04	1.42
		Minimum	0.05	0.13	0.15	0.06	0.07	0.02	0.01
		Maximum	3.32	25.9	4.73	3.10	4.23	0.07	6.79
	Enzymolysis	DR (%)	78.3	96.8	53.3	59.4	75.0	71.8	53.1
		Median	1.56	6.86	25.8	1.65	1.84	1.27	4.97
		Mean	2.81	11.1	41.1	2.83	2.12	1.10	8.69
		Minimum	0.07	1.58	0.21	0.13	0.08	0.01	0.28
		Maximum	19.9	40.7	131	14.6	7.23	1.68	34.3
All samples	Non-enzymolysis	DR (%)	37.9	78.8	44.1	23.3	16.7	6.10	33.9
		Median	0.27	2.69	2.41	0.55	0.47	0.03	1.03
		Mean	0.98	3.95	2.99	1.10	0.83	0.04	2.70
		Minimum	0.01	0.01	0.06	0.001	0.01	0.01	0.01
		Maximum	9.84	94.1	20.9	15.2	4.23	0.08	33.7
	Enzymolysis	DR (%)	71.1	97.1	42.8	48.9	65.7	64.9	33.9
		Median	1.51	4.33	11.9	2.14	1.61	1.28	2.76
		Mean	2.04	8.56	24.1	4.54	1.79	1.05	5.33
		Minimum	0.01	0.03	0.20	0.01	0.01	0.01	0.14
		Maximum	19.9	162	138	37.5	8.79	3.12	34.3

DR—detection rate.

**Table 2:** EDI (ng/kg bw/day) and HQ value of ∑_7_BPs in edible aquatic products of different genders.

Species	Risk Assessment	Male	Female
*Metapenaeus ensis*	50% quantile of EDI	13.34	18.83
	95% quantile of EDI	78.81	93.26
	HQ	0.0015	0.0018
*Aristichys nobilis*	50% quantile of EDI	24.95	25.03
	95% quantile of EDI	97.12	114.9
	HQ	0.0019	0.0022
*Oreochromis mossambicus*	50% quantile of EDI	9.25	23.79
	95% quantile of EDI	104.9	124.2
	HQ	0.0020	0.0024
*Paphia undulata*	50% quantile of EDI	16.85	220.2
	95% quantile of EDI	86.35	102.1
	HQ	0.0017	0.0020
*Trachinotus ovatus*	50% quantile of EDI	18.47	16.86
	95% quantile of EDI	63.76	75.45
	HQ	0.0012	0.0015
*Lateolabrax maculatus*	50% quantile of EDI	14.97	21.12
	95% quantile of EDI	78.62	93.03
	HQ	0.0015	0.0018
*Ctenopharyngodon idella*	50% quantile of EDI	11.43	19.73
	95% quantile of EDI	88.74	105.0
	HQ	0.0017	0.0021
*Carassius auratus*	50% quantile of EDI	16.35	16.86
	95% quantile of EDI	157.4	186.3
	HQ	0.0031	0.0037
All samples	50% quantile of EDI	11.73	16.86
	95% quantile of EDI	104.6	123.7
	HQ	0.0020	0.0024
	The Hazard Index	0.0146	0.0175

EDI: estimated daily intake; HQ: hazard quotient; the Hazard Index (summing HQs).

## Data Availability

Data are available from the corresponding author by request.

## Supplementary Materials

The following supporting information can be downloaded at: https://www.mdpi.com/article/10.3390/toxics12020154/s1, Figure S1: Average concentration distribution of free and bound BPs in aquatic products; Table S1: Mobile phase and gradient elution program; Table S2: Mass spectrometric information of the target compounds; Table S3: Linear equations and related quality control indicators for each analyte; Table S4: Test the difference of concentration levels of BPs in aquatic products with or without enzymatic hydrolysis.
