# Supplementary material for: Bisphenols in Aquatic Products from South China: Implications for Human Exposure
Source: Toxics. 2024 Feb 16;12(2):154. doi: 10.3390/toxics12020154 (PMC10891950; doi:10.3390/toxics12020154)
Supplement: Supplementary file 1 [file toxics-12-00154-s001.zip › toxics-2791225-supplementary.pdf]

Supplementary materials

Bisphenols in aquatic products from South China: Implications for human exposure

Yinhai Chen [1,*], Xiurong Chen [1], Wenchi Lin [1], Jinghong Chen [1], Yuejun Zhu [1] and Zhang-hong Guo [1]

[1] Center for Disease Control and Prevention of Shantou, Shantou China 515041; 13829450098@163.com (X.C.); zhguo2889@163.com (Z.G.)
* Correspondence: stcdcagsea@163.com; Tel.: +86-137-1991-5229

Number of pages (including this page): 6
Number of tables: 4
Number of figures: 1

Table S1. Mobile phase and gradient elution program.

Time (min)	Mobile phase A (%)	Mobile phase B (%)
0	80.0	20.0
1.0	80.0	20.0
12.0	5.0	95.0
13.0	95.0	5.0
14.0	95.0	5.0
15.0	95.0	5.0

Table S2. Mass spectrometetric information of the target compounds.

Chemicals	Precursor ion (*m*/*z*)	Product ion (*m*/*z*)	Dwell (msec)	DP (V)	CE (V)	EP (V)	CXP (V)
BPA	227.10	212.10	25.00	-90.00	-24.00	-10.00	-13.00
	227.10	92.00	25.00	-90.00	-35.00	-10.00	-13.00
BPS	249.20	108.10	25.00	-100.00	-36.00	-10.00	-13.00
	249.20	156.00	25.00	-100.00	-29.00	-10.00	-13.00
BPF	199.00	92.80	25.00	-80.00	-28.00	-10.00	-13.00
	199.00	77.00	25.00	-80.00	-33.00	-10.00	-13.00
BPP	345.00	330.00	25.00	-90.00	-30.00	-10.00	-13.00
	345.00	92.00	25.00	-90.00	-62.00	-10.00	-13.00
BPB	241.00	211.00	25.00	-90.00	-35.00	-10.00	-13.00
	241.00	225.00	25.00	-90.00	-25.00	-10.00	-13.00
BPZ	267.00	173.00	25.00	-100.00	-36.00	-10.00	-13.00
	267.00	223.00	25.00	-100.00	-43.00	-10.00	-13.00
BPAF	335.00	265.00	25.00	-100.00	-30.00	-10.00	-13.00
	335.00	69.20	25.00	-100.00	-70.00	-10.00	-13.00
BPAP	289.00	274.00	25.00	-90.00	-35.00	-10.00	-13.00
	289.00	211.00	25.00	-90.00	-35.00	-10.00	-13.00
BPA-$^{13}C_{12}$	239.00	224.00	25.00	-100.00	-25.00	-10.00	-13.00
BPAF-$^{13}C_{12}$	347.00	277.10	25.00	-15.00	-28.00	-10.00	-13.00
BPB-$^{13}C_{12}$	253.10	223.90	25.00	-180.00	-42.00	-10.00	-13.00
BPF-$^{13}C_{12}$	208.90	97.10	25.00	-100.00	-32.00	-10.00	-13.00
BPS-$^{13}C_{12}$	261.10	113.90	25.00	-170.00	-36.00	-10.00	-13.00

DP: Declustering voltage; CE: Collision energy; EP: Injection voltage; CXP: Injection voltage from collision chamber.

Table S3. Linear equations and related quality control indicators for each analyte.

Analyte	Linear equations	Linearity (R^2)	LOD (ng/g)	LOQ (ng/g)	Linearity range (μg/L)	Recovery (%)
BPA	Y=0.9994x+0.0267	0.999	0.0437	0.230	0.1-50	91.1
BPS	Y=1.0024x-0.0909	0.997	0.0022	0.007	0.1-50	84.8
BPF	Y=1.0096x-0.4314	0.999	0.0780	0.257	0.1-50	87.2
BPP	Y=1.0817x-0.01	0.999	0.0700	0.230	0.1-50	107
BPB	Y=1.0012x-0.0524	0.999	0.0690	0.240	0.1-50	111
BPZ	Y=1.0899x-0.4142	0.999	0.0130	0.052	0.1-50	94.9
BPAF	Y=0.9983x+0.0651	0.999	0.0900	0.300	0.1-50	97.7
BPAP	Y=1.073x+0.2455	0.997	0.0120	0.040	0.1-50	99.7

LOQ: limits of quantification; LOD: limits of detection.

Table S4. Test the difference of concentration levels of BPs in aquatic products with or without enzymatic hydrolysis.

Testing Statistic	BPA	BPS	BPF	BPP	BPZ	BPAP	BPB
Mann-Whitney U test	15311	16949	27166	20250	13588	11335	28829
Wilcoxon-W	45201	47084	57301	50385	43723	41470	58719
rank	-9.800	-8.290	-1.927	-7.245	-11.74	-13.93	-0.806
p value	0.000	0.000	0.050	0.000	0.000	0.000	0.420

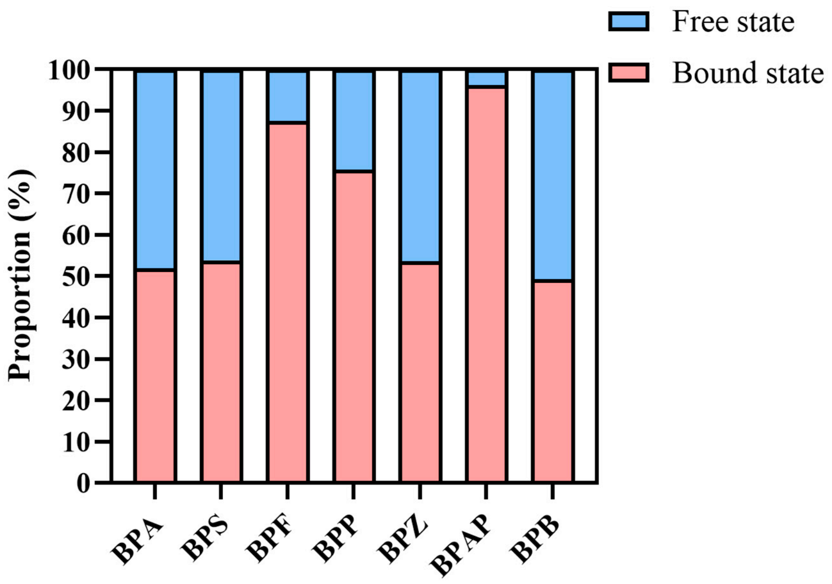

Figure S1. Average concentration distribution of free and bound BPs in aquatic products.
